# Complete Genome Sequence of Staphylococcus capitis CCSM0123, Isolated from Healthy Facial Skin

**DOI:** 10.1128/mra.00132-23

**Published:** 2023-03-14

**Authors:** Laiji Ma, Hong Jiang, Suzhen Yang, Yan Li, Li Shao

**Affiliations:** a School of Perfume and Aroma Technology, Shanghai Institute of Technology, Shanghai, People’s Republic of China; b R&D Innovation Center, Shandong Freda Biotech Co., Ltd., Jinan, Shandong, People’s Republic of China; University of Rochester School of Medicine and Dentistry

## Abstract

Staphylococcus capitis strain CCSM0123 was isolated from healthy facial skin. The complete genome of CCSM0123 was sequenced using a combination of Pacific Biosciences (PacBio) RS II single-molecule real-time (SMRT) and Illumina sequencing. The assembled 2.5-Mbp genome consisted of one chromosome and four plasmids.

## ANNOUNCEMENT

Staphylococcus capitis is a commensal skin bacterium that belongs to the coagulase-negative staphylococci (CoNS). S. capitis can protect against and prevent pathogens, as well as participating in skin equilibrium in their commensal form with the secretion of beneficial metabolites (1–5). S. capitis CCSM0123 was isolated from the healthy facial skin of a Chinese man.

In 2021, a skin microbial sample was collected from the cheek of a healthy man in Shanghai, China (ethics approval number 2021-KY-15; Shanghai Jiao Tong University Affiliated Sixth People’s Hospital South Campus), with a sterile cotton swab and sterile collection solution containing 0.9% NaCl and 0.1% Tween 20. The sample was mixed, serially diluted with sterile water, and then cultured on a tryptic soy agar (TSA) plate containing 5% defibrated sheep blood. After aerobic incubation at 37°C for 12 to 16 h, white round colonies were picked out and then restreaked at least three times on TSA plates to obtain pure cultures, which were stored at −80°C in 30% glycerol; 16S rRNA gene sequencing was carried out using the universal primers 27F and 1492R.

CCSM0123 was activated on TSA plates and then cultured aerobically in tryptic soy broth (TSB) at 37°C for 12 to 16 h with shaking. Genomic DNA was extracted from CCSM0123 using the Wizard genomic DNA purification kit (Promega). Purified genomic DNA was quantified with a TBS-380 fluorometer (Turner BioSystems Inc., Sunnyvale, CA) and sequenced using a combination of Pacific Biosciences (PacBio) RS II single-molecule real-time (SMRT) and Illumina sequencing platforms. For Illumina sequencing, at least 1 μg genomic DNA was used for each strain for sequencing library construction. DNA samples were sheared into 400- to 500-bp fragments using a Covaris M220 focused acoustic shearer following the manufacturer’s protocol. Illumina sequencing libraries were prepared from the sheared fragments using the NEXTFLEX rapid DNA-sequencing kit. Paired-end sequencing libraries (2 × 150 bp) were prepared using a NovaSeq 6000 S4 reagent kit v1.5 (300 cycles) and then were sequenced on an Illumina NovaSeq 6000 system. Raw data from sequencing were stored in fastq format, and fastp v0.23.0 (6) was used to trim raw data according to quality, which yielded 1,179,302,450 bp of raw bases, with 150-bp read length; clean reads with scores of Q20 accounted for 97.76% of all reads. For PacBio sequencing, an aliquot of 15 μg DNA was centrifuged in a Covaris g-TUBE at 6,000 rpm for 60 s using an Eppendorf 5424 centrifuge, which processed genomic DNA into ~10-kb fragments. DNA fragments were then purified, end repaired, and ligated with SMRTbell sequencing adapters following the manufacturer’s recommendations (PacBio, San Diego, CA), which yielded 787,277 reads with an average length of 10,835.52 bases and an *N*_50_ value of 11,467bp.

The Illumina and PacBio read data were assembled *de novo* using Unicycler v0.4.8 (7) to yield structurally consistent assembled sequences, which were then iteratively polished with Pilon v1.22 (8) to generate a single circular chromosome of 2,453,862 bp (GC content, 32.88%) and four circular plasmids, i.e., plasmid A (48,504 bp), plasmid B (35,327 bp), plasmid C (7,369 bp), and plasmid D (3,982 bp). Rotation and circularity were confirmed via Unicycler (Table 1). Glimmer v3.02 (9) was used for coding sequence (CDS) prediction, tRNAscan-SE v2.0 (10) was used for tRNA prediction, and Barrnap v0.9 (11) was used for rRNA prediction; 2,472 CDSs, 19 rRNAs, 61 tRNAs, and 60 small RNAs were identified. Default parameters were used for all software unless otherwise specified.

**TABLE 1:** Genome statistics and features of Staphylococcus capitis strain CCSM0123

Chromosome or plasmid	Length (bp)	GC content (%)	No. of CDSs	No. of rRNAs	No. of tRNAs	GenBank accession no.
Chromosome	2,453,862	32.88	2,472	19	61	CP110784
Plasmid A	48,504	31.59	53	0	0	CP110785
Plasmid B	35,327	29.57	28	0	0	CP110786
Plasmid C	7,369	31.25	9	0	0	CP110787
Plasmid D	3,982	29.48	4	0	0	CP110788

### Data availability.

The complete genome sequence of S. capitis CCSM0123 is available in GenBank under the accession number CP110784.1, BioProject accession number PRJNA894108, BioSample accession number SAMN31437481, and Sequence Read Archive (SRA) accession number SRS16733147. S. capitis strain CCSM0123 has been deposited in the China Center for Type Culture Collection under accession number CCTCC M2022771.
